# Roles of Omega-3 Polyunsaturated Fatty Acids in Managing Cognitive Impairment in Chronic Obstructive Pulmonary Disease: A Review

**DOI:** 10.3390/nu15204363

**Published:** 2023-10-13

**Authors:** Halliru Zailani, Senthil Kumaran Satyanarayanan, Wei-Chih Liao, Yi-Ting Hsu, Shih-Yi Huang, Piotr Gałecki, Kuan-Pin Su, Jane Pei-Chen Chang

**Affiliations:** 1 Mind-Body Interface Laboratory (MBI-Lab), Department of Psychiatry, China Medical University Hospital, Taichung 404327, Taiwan; halliruzyln55@gmail.com (H.Z.); talakokkalu@gmail.com (S.K.S.); 2 Graduate Institute of Nutrition, China Medical University, Taichung 404, Taiwan; 3 Department of Biochemistry, Ahmadu Bello University, Zaria 810106, Nigeria; 4 Division of Pulmonary and Critical Medicine, Department of Internal Medicine, China Medical University Hospital, Taichung 404327, Taiwan; 5 Department of Neurology, China Medical University Hospital, Taichung 404327, Taiwan; d11835@mail.cmuh.org.tw; 6 School of Nutrition and Health Sciences, Taipei Medical University, Taipei 11031, Taiwan; 7 Nutrition Research Centre, Taipei Medical University Hospital, Taipei 110, Taiwan; 8 Department of Adult Psychiatry, Medical University of Lodz, 91-229 Lodz, Poland; piotr.galecki@umed.lodz.pl; 9 College of Medicine, China Medical University, Taichung 404, Taiwan; 10 Graduate Institute of Biomedical Sciences, China Medical University, Taichung 404, Taiwan; 11 An-Nan Hospital, China Medical University, Tainan 717, Taiwan

**Keywords:** Alzheimer’s disease, chronic obstructive pulmonary disease, cognitive impairment, omega-3 polyunsaturated fatty acids, Parkinson’s disease

## Abstract

Chronic obstructive pulmonary disease (COPD) contributes significantly to the death of people worldwide, especially the elderly. An essential feature of COPD is pulmonary inflammation, which results from long-term exposure to noxious substances from cigarette smoking and other environmental pollutants. Pulmonary inflammatory mediators spill over to the blood, leading to systemic inflammation, which is believed to play a significant role in the onset of a host of comorbidities associated with COPD. A substantial comorbidity of concern in COPD patients that is often overlooked in COPD management is cognitive impairment. The exact pathophysiology of cognitive impairment in COPD patients remains a mystery; however, hypoxia, oxidative stress, systemic inflammation, and cerebral manifestations of these conditions are believed to play crucial roles. Furthermore, the use of medications to treat cognitive impairment symptomatology in COPD patients has been reported to be associated with life-threatening adverse effects, hence the need for alternative medications with reduced side effects. In this Review, we aim to discuss the impact of cognitive impairment in COPD management and the potential mechanisms associated with increased risk of cognitive impairment in COPD patients. The promising roles of omega-3 polyunsaturated fatty acids (ω-3 PUFAs) in improving cognitive deficits in COPD patients are also discussed. Interestingly, ω-3 PUFAs can potentially enhance the cognitive impairment symptomatology associated with COPD because they can modulate inflammatory processes, activate the antioxidant defence system, and promote amyloid-beta clearance from the brain. Thus, clinical studies are crucial to assess the efficacy of ω-3 PUFAs in managing cognitive impairment in COPD patients.

## 1. Introduction

### 1.1. Chronic Obstructive Pulmonary Disease

Chronic obstructive pulmonary disease (COPD) is a lung-related inflammatory condition primarily caused by prolonged exposure to harmful substances in the environment, particularly cigarette smoke and other toxic gases [1]. COPD affects over 380 million people globally and is linked to increased healthcare utilisation, decreased quality of life, and higher mortality rates [2,3,4]. In 2019, COPD was responsible for over 3 million deaths, with the majority occurring in low- and middle-income countries [4]. The critical symptoms of COPD include persistent coughing and irreversible airflow restriction, resulting in breathlessness [5]. Additionally, COPD is associated with recurrent periods of worsened lung function, known as exacerbation, primarily triggered by exposure to harmful microbes, like bacteria and viruses, in the environment [6]. Exacerbation in COPD is a common cause of morbidity and mortality related to the condition [6]. In addition to its primary effect on the lungs, COPD is associated with a host of extrapulmonary manifestations.

Inflammation is a crucial clinical aspect of COPD. Exposure to harmful substances from cigarette smoke and other environmental pollutants may lead to pulmonary inflammation. Immune cells become activated when exposed to oxidants from cigarette smoke, producing reactive oxygen and nitrogen species, which lead to oxidative stress. This oxidative stress activates proinflammatory factors, like nuclear kappa beta (NF-κβ), leading to local inflammation. Notably, higher levels of inflammatory cells, such as alveolar macrophages, neutrophils, and T lymphocytes, have been observed in individuals with COPD [7]. These cells release inflammatory mediators, including proinflammatory cytokines, contributing to pulmonary inflammation. The accumulated inflammation markers in the lungs of COPD patients may seep into peripheral tissues, leading to systemic inflammation. Indeed, substantial elevations in inflammatory markers, like C-reactive protein (CRP), leukocytes, interleukin (IL)-6, IL-8, fibrinogen, and tumour necrosis factor (TNF)-α, have been reported in COPD patients when compared to healthy individuals [8,9]. 

Systemic inflammation has been widely theorised to play a pivotal role in the development of comorbidities beyond the lungs, including mental health issues, like depression [10] and cognitive impairment (CI) [11], in COPD patients. Specifically, inflammatory mediators, including IL-6, TNF-α, and IL-1β, have been shown to breach the central nervous system (CNS) by disrupting the blood–brain barrier (BBB), leading to neuroinflammation [12]. Neuroinflammation, in turn, can damage neurons and impair neuronal functions, which may contribute to CI [13]. Hypoxia, characterised by inadequate oxygen supply in tissues, is also believed to increase the susceptibility of COPD patients to CI. COPD is marked by insufficient airflow in the lungs; thus, hypoxia is common. Hypoxia hinders the production of critical neurotransmitters, such as dopamine and serotonin, in the brain [14] because the essential enzymes responsible for the production of these neurotransmitters rely on oxygen for optimal function [14,15].

CI in COPD patients has far-reaching adverse effects on both COPD clinical management and the quality of life of affected individuals [16,17]. Despite its debilitating impact on COPD management, CI is often overlooked or undiagnosed, receiving limited attention in COPD care. Moreover, pharmacological drugs used to address CI symptoms in COPD patients are associated with various health complications, underscoring the need for alternative therapies with minimal side effects. Increasing evidence suggests that diet, mainly one that is rich in omega-3 polyunsaturated fatty acids (ω-3 PUFAs), plays a significant role in brain health [18,19] and may be beneficial in managing CI in COPD patients [19].

### 1.2. ω-3 PUFAs

ω-3 PUFAs are essential fatty-acid types characterised by multiple double bonds in their carbon chain. They are deemed essential because the human body lacks the enzymes to produce them endogenously, making obtaining them from external sources crucial. Alpha-linolenic acid (ALA) is the simplest form of ω-3 PUFA and is commonly found in flaxseed and, to a lesser extent, in soybean and canola oils [20]. When consumed, ALA is converted into more physiologically active ω-3 PUFAs, known as eicosapentaenoic acid (EPA) and docosahexaenoic acid (DHA), through a series of enzyme-catalysed reactions involving elongases and desaturases [21]. However, this conversion rate is very limited in humans, with a 7–21% conversion rate for EPA and a 0.01–1% conversion rate for DHA [21]. As the EPA and DHA endogenous synthesis from ALA is insufficient to meet the body’s physiological needs, these important ω-3 PUFAs can be chiefly obtained from external sources, like fish and fish oils. Indeed, a high intake of ω-3 PUFAs has been linked with a decreased likelihood of developing conditions, such as cardiovascular disorders [22], major depressive and anxiety disorders [23,24], and neurodegenerative disorders [25,26]. Additionally, there has been extensive discussion about the potential of ω-3 PUFAs in managing comorbid depression in COPD patients [10]. 

However, despite the recognised health benefits of ω-3 PUFAs for brain health and cognition, there is a general lack of studies examining their effects on managing CI in COPD patients. Therefore, this Review will discuss the impacts of comorbid CI on COPD management, the potential mechanisms behind the heightened CI associated with COPD, and the potential of ω-3 PUFAs in managing comorbid CI in COPD patients.

## 2. Comorbid Cognitive Impairment in COPD Patients

CI is a significant comorbidity often seen in COPD patients [27]. A growing body of evidence indicates a notable occurrence of CI in individuals with COPD [28,29,30]. CI can vary in severity, ranging from mild to severe. Among the severe forms of CI, Alzheimer’s disease and Parkinson’s disease are the two most encountered in the COPD population.

### 2.1. Mild Cognitive Impairment and Alzheimer’s Disease 

The relationship between CI and COPD has been extensively studied. Indeed, studies have reported mild CI and Alzheimer’s disease as significant comorbid conditions in COPD patients. For instance, a study showed that approximately one-third (36%) of COPD patients experience mild CI, a higher prevalence when compared to that found among healthy individuals (12%) [31]. Similarly, poorer performances in various neuropsychological tests, such as Raven’s Coloured Progressive Matrices, Trail-Making Parts A and B, visual search, story recall, and phonological and semantic fluency, have been reported among COPD patients (*n* = 22) when compared to age- and gender-matched healthy controls (*n* = 22) [32]. Furthermore, COPD patients are 1.74-fold more likely to develop Alzheimer’s disease when compared to individuals without COPD, according to a large retrospective study involving 8640 COPD patients and 17,280 controls [33]. Another study reported a 1.27-fold increased risk of Alzheimer’s disease in COPD patients after accounting for other coexisting conditions [34]. Similarly, a large national cohort study, including COPD and asthma patients (10,260 participants) and healthy controls (20,513 participants), reported a 1.43-times increased risk of Alzheimer’s disease in individuals with COPD when compared to those in the control group [35]. Furthermore, the onset of COPD in midlife has been linked to a 1.85-fold increased risk of CI and dementia later in life [36]. CI in COPD patients has been shown to correlate with COPD acute exacerbations. Specifically, patients with acute exacerbations of COPD exhibited more severe CI than those with stable COPD [27]. Additionally, a stronger association with Alzheimer’s disease was observed in COPD patients who experienced frequent acute exacerbations [34]. 

The presence of coexisting mild CI and Alzheimer’s disease in COPD patients negatively impacts disease management and outcomes [27]. COPD patients with CI reported lower health-related qualities of life (HRQoLs), increased mortality rates, and higher rates of rehospitalisation when compared to those without CI [37]. Additionally, COPD patients with CI may face challenges in adhering to therapy and self-managing their condition, as CI has been associated with the inability to carry out memory-intensive tasks [38]. Alzheimer’s disease can worsen the severity of COPD, leading to poorer clinical outcomes and higher mortality rates among COPD patients. COPD patients with Alzheimer’s disease may find it challenging to adhere to treatment guidelines and perform memory-intensive tasks, such as using inhalers [39] and performing self-care activities [17], which may predispose these patients to an increased risk of frequent exacerbations. Moreover, COPD patients with Alzheimer’s disease face a significantly higher risk of developing acute respiratory dysfunction, severe sepsis, and hospital mortality when compared to those without Alzheimer’s disease [40]. Therefore, the management of CI in COPD patients is crucial for improving hospital outcomes and the quality of life of COPD patients. The symptomatic treatment of CI and Alzheimer’s disease often involves antipsychotic drugs when necessary. However, antipsychotic drug use in COPD patients has been associated with a 1.66-fold increased risk of acute respiratory failure, according to a recent study [41]. Acetylcholinesterase inhibitors are frequently prescribed to Alzheimer’s disease patients to enhance their acetylcholine levels. Nonetheless, the use of these drugs to treat dementia in COPD patients has been linked to a higher frequency of exacerbations in the initial three months of use [41]. In summary, these side effects associated with the use of antipsychotic drugs and acetylcholinesterase inhibitors highlight the need for alternative medications with more manageable side effects for use in CI in COPD patients.

### 2.2. Parkinson’s Disease

Parkinson’s disease is a neurological condition marked by motor symptoms and is the second most common neurodegenerative disorder after Alzheimer’s disease [42]. The connection between Parkinson’s disease and COPD has not been extensively studied. A large national cohort study found that COPD patients (*n* = 20,728) had a 1.73-times higher likelihood of developing Parkinson’s disease when compared with healthy controls (*n* = 41,147), even after accounting for gender, age, and other existing health conditions [43]. Additionally, a higher occurrence of Parkinson’s disease was observed in COPD patients who had other health issues, like coronary artery disease, stroke, hyperlipidaemia, hypertension, and head injury, in the study [43]. Parkinson’s disease in addition to COPD may worsen the patient’s condition, especially by heightening anxiety and depressive symptoms. This is significant, as elevated rates of anxiety and depression are also reported in individuals with Parkinson’s disease [44,45]. In COPD patients, heightened anxiety and depression may be associated with poorer disease management, higher mortality rates, reduced quality of life, increased rehospitalisation, and prolonged hospital stays [46,47,48]. Moreover, the memory impairment linked with Parkinson’s disease might make it challenging for COPD patients to adhere to their prescribed COPD management plans [49]. Even though pulmonary rehabilitation (PR) involving exercise training is vital for enhancing respiratory health and airflow in COPD patients, comorbid Parkinson’s disease might hinder patients from actively participating in PR owing to the motor difficulties associated with Parkinson’s disease.

Currently, no pharmacological drugs can provide a definitive cure for Parkinson’s disease. Many potential treatments that initially showed promise in pre-clinical studies failed to demonstrate effectiveness in clinical trials [50]. Consequently, present therapies are focused on alleviating the symptoms connected with Parkinson’s disease. Levodopa (L-DOPA) is currently the most efficacious drug in managing Parkinson’s disease [51]. Indeed, a study has shown that L-DOPA enhances pulmonary functions in PD patients [51]. However, there is a lack of studies on the effectiveness of L-DOPA in COPD patients, so its efficacy in this condition is yet to be established. It is worth noting that the use of L-DOPA has been associated with respiratory difficulties in a Parkinson’s disease case with COPD [52].

## 3. Possible Biological Links between COPD and Cognitive Impairment

The exact link between COPD and CI remains elusive. However, it has been theorised that the manifestation of CI in COPD patients may result from some shared mechanisms or risk factors between COPD and CI. These mechanisms may be related to inflammation, oxidative stress, amyloid-β (Aβ) accumulation, cigarette smoking, hypoxia, and gut dysbiosis.

**Figure 1:**
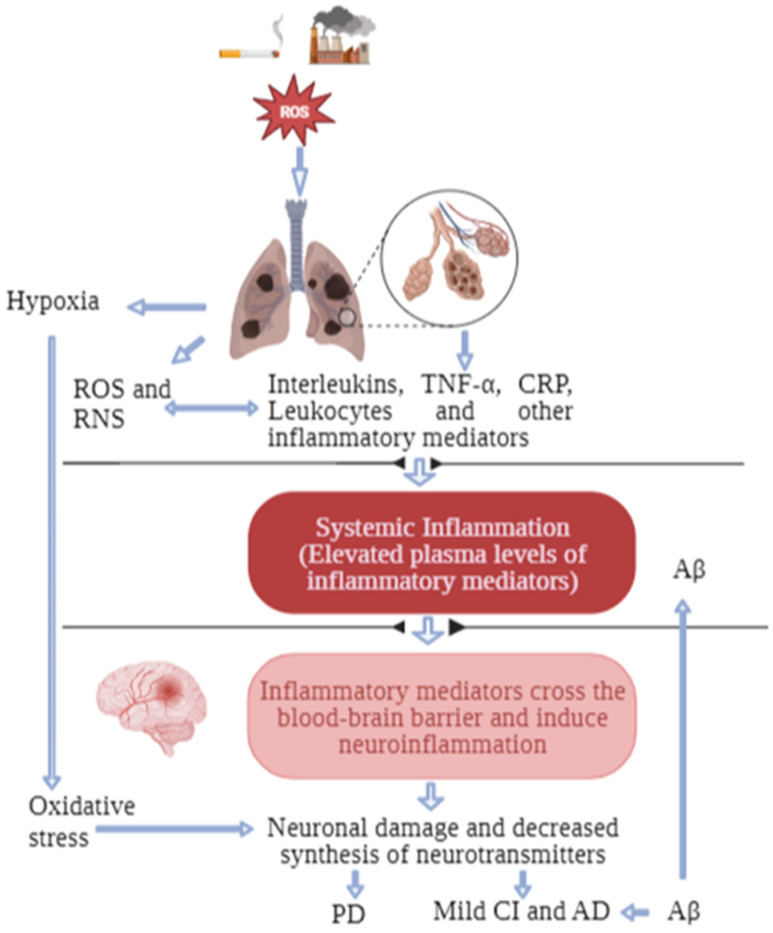
Possible mechanisms for increased risk of CI in patients with COPD Owing to cigarette smoke and other noxious environmental substances, reactive oxygen species accumulate in the lungs and trigger local inflammation. The inflammatory mediators in the lungs spill into the plasma and cause systemic inflammation. Inflammatory mediators cross the blood–brain barrier and cause neurodegeneration and reduced synthesis of neurotransmitters, resulting in PD, CI, and AD. Hypoxia also leads to PD due to reduced synthesis of dopamine in the brain. The accumulation of Aβ in the plasma of COPD patients accelerates its deposition in the brain, thus predisposing them to AD and CI. AD: Alzheimer’s disease; Aβ: amyloid-beta; CI: cognitive impairment; COPD: chronic obstructive pulmonary disease; CRP: C-reactive protein; PD: Parkinson’s disease; TNF-α: tumour necrosis factor-alpha.

COPD is characterised by systemic inflammation [7] (Figure 1), which is widely believed to originate from the leakage of inflammatory mediators from the lungs into the circulatory system [10]. Previous studies have shown that patients with COPD have higher levels of serum inflammatory cytokines when compared to healthy controls [8,53]. Furthermore, inflammatory cytokines, such as TNF-α, IL-1β, and IL-6, have been demonstrated to be associated with COPD severity [54,55]. On the other hand, systemic inflammation is also a critical pathophysiological phenomenon in CI [56]. Elevations in circulating levels of proinflammatory cytokines have been reported in patients with CI. A meta-analysis showed higher peripheral levels of IL-6, TNF-α, IL-1β, IL1-2, IL-18, and transforming growth factor (TGF)-β in patients with Alzheimer’s disease [57]. Studies have also shown a positive association between the concentration of high-sensitivity (hs)-CRP and mild CI [58] and between both CRP and IL-6 and a decline in cognitive abilities and executive functions [59]. Inflammatory cytokines associated with COPD can cross the BBB and induce neuroinflammation, triggering neuronal damage and a decline in neuronal functions [60]. Additionally, in the peripheral tissues, inflammatory cytokines, such as interferons, have been reported to increase the degradation of tryptophan via the kynurenine pathway, which may hinder the adequate transport of tryptophan to the brain for synthesising neurotransmitters that are crucial for cognition [10]. The link between inflammation and brain-related comorbidities in COPD patients has been well discussed [10,11]. However, studies on the relationship between inflammation and CI in COPD patients are generally scarce. A study reported a negative correlation between serum CRP levels and CI in COPD patients [61]. In another study, serum monocyte chemoattractant protein-1 was reported to negatively correlate with cognitive functions in patients with COPD and comorbid obstructive sleep apnoea-hypopnea syndrome [62]. Similarly, a recent study reported an increased serum Aβ level associated with disease severity in cognitively normal COPD patients compared to normal controls [63]. Furthermore, the Aβ levels were significantly higher in COPD patients with more highly elevated CRP and IL-6 levels [63]. This finding suggests that inflammation in COPD patients may trigger Alzheimer’s disease-related pathogenesis, which may further lead to cognitive decline in this population.

Cigarette smoking, the leading risk factor for COPD, could further increase the risk of CI in COPD patients via its influence on the BBB, leading to neuroinflammation. The BBB plays a crucial role in controlling the movement of molecules into and out of the brain. An impaired BBB will allow the entry of neurotoxic blood-derived debris, cells, and microbial pathogens to the brain, which are associated with several immune and inflammatory responses, leading to neurodegeneration [64]. In the case of COPD, inflammatory mediators can disrupt the integrity of the BBB, leading to cognitive dysfunction. Indeed, it has been demonstrated that the BBB is compromised in CI and Alzheimer’s disease [64,65] and may be strongly related to the severity of these diseases. In vitro studies have shown that the treatment of human BBB endothelial cells with cigarette-smoke extract disrupts the endothelial cells and decreases the expression of tight junction proteins, such as claudin-5, occludin, and ZO-1 [66,67]. Additionally, mice that were exposed to cigarette smoke and lipopolysaccharide (LPS) to induce COPD-like features were found to have reduced expression of claudin-5 and occludin in brain micro-vessels and increased microglial activation in the hippocampal region of the brain [68]. These findings suggest that cigarette smoke and LPS induce neuroinflammation by increasing the BBB permeability, partially owing to the oxidative stress caused by the free radicals in cigarette smoke [69]. Moreover, oxidants from cigarette-smoke exposure trigger an immune response, resulting in elevated levels of proinflammatory cytokines, e.g., TNF-α and IL-6 [70,71,72], which can directly impair the integrity and functions of the BBB. Furthermore, the weakened tight junctions allow the entry of innate immune cells from the systemic circulation through the BBB to the brain tissue, thus triggering inflammatory reactions and, ultimately, neuroinflammation [73]. Aβ deposition in the brain is a central mechanism in the pathogenesis of Alzheimer’s disease [74]. A recent case-control study discovered a correlation between cigarette smoking and elevated levels of Alzheimer’s disease risk indicators; elevated Aβ42 levels, increased oxidative-stress markers, neuroinflammation, and reduced neuroprotection in the cerebrospinal fluid (CSF) of participants who were active smokers [75].

Neuroinflammation pertains to localised inflammatory reactions occurring within the CNS, primarily driven by the release of cytokines, chemokines, and reactive oxygen species (ROS) from critical immune cells in the CNS, known as microglia and astrocytes [76,77]. Microglia, the resident immune cells in the white and grey matter of the brain, constitute roughly 10% of the CNS population [78]. Under ideal conditions, microglial cells are considered to be in a surveillance state, actively scanning the CNS for foreign materials, such as pathogens [79]. Upon encountering a stimulus or injury, microglia extend their processes toward the site of damage and eliminate foreign materials through phagocytosis [80]. Although acute microglial activation is beneficial under pathological conditions, prolonged activation of these cells can lead to detrimental effects, including neuronal damage [81] and impaired cognition. A meta-analysis of 28 studies consisting of participants with mild CI (*n* = 168), Alzheimer’s disease (*n* = 269), and healthy controls (*n* = 318) showed a significant increase in the levels of translocator protein (TSPO), indicating microglial activation [82], in the brains of subjects with mild CI and Alzheimer’s disease when compared with the brains of the healthy controls [83]. Moreover, elevated levels of TGF-β, chitinase-3-like 1, and monocyte chemoattractant protein-1 were reported in the CSF of patients with Alzheimer’s disease when compared to healthy controls [84]. Furthermore, increased immune activation, indicated by high levels of inflammatory mediators and activated microglia, was reported in the substantia nigra and striatum of patients with Parkinson’s disease [85,86]. To the best of our knowledge, whether neuroinflammation is associated with CI in COPD patients has not previously been studied. However, a preclinical study demonstrated significant microglial activation after the administration of the tobacco-specific procarcinogen, 4-N-methyl-N-nitrosamino-1-(3-pyridyl)-1-butanone, in BALB/c mice [87]. This finding corroborates a wealth of existing animal-model research indicating that exposure to cigarette smoke and e-cigarettes tends to provoke a proinflammatory response in the brain, often linked with microglial activation [87,88,89]. Furthermore, sustained nicotine administration in rodents was shown to lead to increased microglial activation in the nucleus accumbens, which diminished after acute nicotine withdrawal [90].

Hypoxia occurs when there is insufficient oxygen at the tissue level to maintain tissue homeostasis. One of the symptoms of COPD is difficulty in breathing, which is associated with inflammation in pulmonary airways and results in insufficient oxygen content in the blood (hypoxaemia) and, ultimately, hypoxia. Hypoxia has been shown to impair the synthesis of neurotransmitters in the brain, leading to changes in neuronal functioning and, eventually, CI [10]. Hypoxia may also induce CI via its stimulatory effect on oxidative stress and inflammation. Indeed, hypoxia has been shown to promote ROS production [91], which may trigger inflammation via the activation of NF-kβ [92]. One of the body’s responses to hypoxia is the upregulation of hypoxia-inducible factor (HIF)-1. Under hypoxic conditions, HIF-1 directs the limited oxygen supply in the brain to synthesise neurotransmitters [93]. HIF-1 stimulates dopamine production and the development of dopaminergic neurons [93] and protects dopaminergic neurons by regulating iron homeostasis and enhancing the resilience to oxidative stress and mitochondrial disruption [94,95]. However, individuals with COPD showed a diminished response to hypoxia, linked to lower histone deacetylase 7 (HDAC-7) and HIF-1α levels [96], which may trigger hypoxia-induced CI in COPD patients.

The relationship between hypoxia and CI in COPD patients has been widely studied; however, the findings have been inconsistent. Several studies have found a significant correlation between hypoxaemia and CI in COPD patients. Notably, low oxygen saturation in COPD patients was positively associated with an increased risk of CI (OR 5.45) [97]. Furthermore, the frequent use of oxygen therapy has significantly reduced the risk of CI in COPD patients [97]. In another study, Karamanli et al. found that long-term oxygen therapy-dependent (LTOTD) COPD patients demonstrated a significantly higher cognitive status when compared with non-LTOTOD COPD patients [98]. On the other hand, CI was reported in COPD patients with and without hypoxaemia [99,100]. Neuroimaging studies have revealed that hypoxemic COPD patients exhibit decreased hippocampal volume [101] and signs of cerebral perfusion [102,103] when compared to normal and non-hypoxemic COPD patients. Conversely, a study reported hippocampal shrinkage and significant decreases in white matter integrity and grey matter functional activation in stable COPD patients with no signs of hypoxaemia [27]. These mixed findings suggest that cognitive dysfunction in COPD patients is not solely attributable to hypoxaemia but a confounding influence of multiple biological mechanisms.

There is emerging evidence that the gut microbiota may also play a role in COPD-induced CI. The gut microbiota is the population of beneficial, non-pathogenic microbes that inhabit the digestive tracts of humans. In physiologically healthy individuals, the gut microbiota provides various advantages, including safeguarding and maintaining the gut and aiding in the absorption of nutrients [104]. It also offers protection against viral diseases [105]. Substantial evidence has shown that a well-diversified gut microbiota is crucial for maintaining good health [106]. The gut microbiota plays an essential role in the connection between the gut and the brain, as it releases metabolic byproducts and produces molecules that trigger physiological changes in the CNS [107]. The two-way communication between the gut microbiota and the brain is highly sensitive to alterations, and external stressors can shift the microbiota’s composition toward an unfavourable microbial community, a condition known as dysbiosis. Dysbiosis has been shown to increase the production of proinflammatory cytokines in the peripheral tissues and CNS [108]. Furthermore, dysbiosis has been indicated to regulate tryptophan availability via the kynurenine pathway, which may impair the optimal transport of tryptophan for synthesising serotonin and may lead to CI [109]. Indeed, gut dysbiosis has been linked to impaired brain functions in many brain diseases, such as Alzheimer’s disease [110], Parkinson’s disease [111], bipolar disorder [112], and major depressive disorder [113]. Recent studies have suggested that cigarette smoke leads to changes in gut dysbiosis in both humans and rodents [114], potentially offering a mechanism for cognitive decline in individuals with COPD. Additionally, Li et al. reported that the gut microbiome of COPD patients significantly varied from that of healthy controls and was characterised by a distinct overall microbial diversity and composition and reduced levels of short-chain fatty acids [115]. It was further demonstrated that compared with healthy controls, COPD patients exhibited 146 different bacterial species in their faecal samples, which were correlated with decreased lung function [116]. Of note, there is a lack of studies on the relationship between gut dysbiosis and CI in COPD patients. Therefore, well-designed epidemiological studies are needed to establish the association between gut dysbiosis and CI in COPD patients.

## 4. Potential Roles of ω-3 PUFAs in COPD and Comorbid Cognitive Impairment

### 4.1. Mild Cognitive Impairment and Alzheimer’s Disease

The evidence suggests that ω-3 PUFAs play a significant role in mental health. Notably, substantial evidence indicates that low ω-3 PUFA levels are associated with many psychiatric disorders, such as depression [117], attention-deficit hyperactivity disorder [118], bipolar disorder [119], and CI [120,121]. Indeed, elderly patients with mild CI and Alzheimer’s disease have notably lower levels of total ω-3 PUFAs and a lower ratio of ω-3 to ω-6 PUFAs when compared to healthy control groups [120]. Additionally, lower plasma levels of ω-3 PUFAs have been linked to poorer cognitive functioning in older adults with CI and Alzheimer’s disease [122]. Furthermore, higher serum levels of EPA have been associated with a reduced incidence of all-cause dementia (HR 0.76) and Alzheimer’s disease (HR 0.66) in the oldest adults [123]. Similarly, individuals with Alzheimer’s disease who had lower baseline levels of DHA were found to have a higher risk of cognitive decline when compared to individuals with higher baseline levels of DHA (OR 1.131) [124]. Furthermore, a longitudinal study of 899 individuals without dementia, at baseline, found that individuals in the highest quartile of plasma phosphatidylcholine DHA levels had a 47% lower likelihood of developing all-cause dementia over ten years [125]. These findings were further supported by a meta-analytic study of 10 studies, where lower plasma levels of ω-3 PUFAs were reported in patients with CI and Alzheimer’s disease when compared to healthy controls [121]. Indeed, ω-3 PUFA deficiency in patients with mild CI and Alzheimer’s disease may be attributable to the suboptimal dietary intake of these crucial nutrients, as studies have indicated that low consumption of ω-3 PUFAs is associated with an increased risk of mild CI and Alzheimer’s disease. For instance, a cohort study by Barberger-Gateau and colleagues involving 80,085 non-demented individuals above age 65 reported a decreased risk of Alzheimer’s disease with frequent intakes of fish and ω-3 PUFAs [126]. Similarly, high consumption of ω-3 PUFAs was linked to a decreased odds ratio of mild CI in a cohort of individuals without dementia [127]. Furthermore, a prospective study conducted between 1993 and 2000 found that the dietary intake of ω-3 PUFAs and weekly fish consumption were associated with a decreased incidence of Alzheimer’s disease [128]. Moreover, regular consumption of ω-3 PUFAs and seafood was reported to offer protection against cognitive decline in a longitudinal study [129]. A meta-analysis of 21 cohort studies further reported negative associations between the intake of fish products and the risks of CI and Alzheimer’s disease [130]. Whether ω-3 PUFA deficiency is associated with CI in COPD patients is currently unknown, as studies in this area are generally lacking, even though COPD is also characterised by a marked deficiency in ω-3 PUFAs [131]. Thus, studies are needed to assess the relationship between ω-3 PUFAs and CI in COPD patients.

The beneficial effects of ω-3 PUFAs on improving cognition have been widely reported in interventional studies. In a clinical trial, ω-3 PUFA supplementation attenuated cognitive decline in individuals with mild Alzheimer’s disease [132]. Additionally, ω-3 PUFAs were associated with significant improvements in short-term working memory, immediate verbal memory, and delayed recall in subjects with mild CI [133]. Bo and colleagues also found that supplementation with ω-3 PUFAs improved the cognitive functions of older adults with mild CI [134]. In another trial, supplementation with fish oil improved cognitive symptoms in older adults with subjective CI [135]. Meta-analytic studies have further supported the beneficial effects of ω-3 PUFAs on CI. Alex and colleagues reported mild but positive impacts of ω-3 PUFA supplementation on memory functions in older adults without dementia [136]. Another meta-analysis noted improved cognitive function in veterans with mild CI who were supplemented with long-chain ω-3 PUFAs when compared to those who received a placebo [137]. Furthermore, in another meta-analysis, DHA monotherapy or combined with EPA contributed to memory functions in older people with mild memory complaints [138].

**Figure 2:**
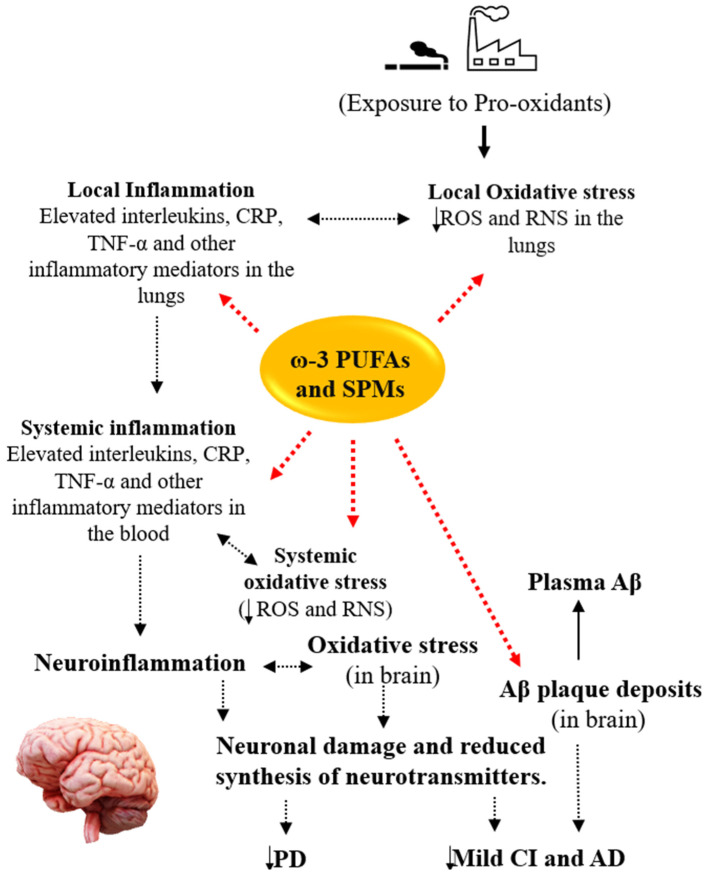
Summary of the potentials of ω-3 PUFAs in managing COPD comorbid with CI. ω-3 PUFAs inhibit inflammatory pathways, activate antioxidant enzymes, improve Aβ clearance from the brain, and block inflammatory mediators from entering the brain. Aβ: amyloid-beta; AD: Alzheimer’s disease; CI: cognitive impairment; COPD: chronic obstructive pulmonary disease; CRP: C-reactive protein; PD: Parkinson’s disease; ω-3 PUFAs: omega-3 polyunsaturated fatty acids; RNS: reactive nitrogen species; ROS: reactive oxygen species; SPMs: specialised pro-resolvin mediators; TNF-α: tumour necrosis factor-alpha.

Indeed, ω-3 PUFAs may benefit COPD individuals with CI through the ability of ω-3 PUFAs to assist in Aβ clearance, modulate inflammation, and boost the body’s antioxidant capacity (Figure 2). The potential of ω-3 PUFAs for clearing Aβ has been studied. In a study, ω-3 PUFAs were shown to significantly enhance the clearance of interstitial Aβ from the brain and protect against Aβ-induced injury [139]. Additionally, supplementation with ω-3 PUFAs has been found to promote Aβ clearance from the brain to the systemic circulation, as evidenced by reduced Aβ levels and fewer senile plaques in the brain parenchyma, along with a simultaneous increase in Aβ levels in the plasma of mouse models of Alzheimer’s disease [140]. Diets with a higher ratio of ω-6 to ω-3 PUFAs have been associated with increased Aβ levels in the brains of male transgenic mouse models of Alzheimer’s disease; however, this increase in Aβ levels was reversed by diets with a higher ratio of ω-3 to ω-6 PUFAs [141]. Moreover, DHA-enriched diets have been shown to significantly decrease the total Aβ and overall plaque burdens in the hippocampus and parietal cortex of the brains in transgenic mice [142]. Both DHA and EPA have been found to enhance the removal of Aβ in human microglial cells [143]. Maresin (MaR), a pro-resolving mediator derived from DHA, has been reported to inhibit the Aβ-induced increase in cytokine secretion and stimulate the uptake of Aβ in both monocyte-derived microglia and differentiated human monocyte cell lines [144]. Indeed, supplementation with ω-3 PUFAs enhanced Aβ phagocytosis by monocytes and increased resolvin D1 (RvD1) levels in patients with mild CI [145]. Moreover, ω-3 PUFAs improved Aβ macrophage-mediated phagocytosis in patients with mild CI [146].

ω-3 PUFAs could help to improve the cognitive functions of COPD patients via their modulatory effect on inflammation. Indeed, inflammation is one of the suggested links between COPD and CI. Notably, a study found that higher intakes of ALA were linked to lower levels of serum TNF-α in 250 stable COPD patients [147]. Conversely, increased intakes of proinflammatory arachidonic acid were associated with higher levels of serum IL-6 and CRP [147]. Another study revealed that supplementation with ω-3 PUFAs, along with lycopene and rosuvastatin, decreased plasma IL-6 levels and restored leukotriene B4 receptor gene expression to its initial levels in COPD patients [148]. Furthermore, COPD patients with cachexia showed lower IL-6, IL-8, and TNF-α levels after receiving high-doses of ω-3 PUFAs, vitamin D, and high-quality protein [149]. Sugawara and colleagues similarly observed lower serum levels of hs-CRP, IL-6, IL-8, and TNF-α in COPD patients after supplementation with a nutritional drink containing ω-3 PUFAs and vitamin A, in addition to engaging in low-intensity exercise [150]. Moreover, a recent meta-analysis reported a reduction in IL-6 levels among COPD patients who were supplemented with ω-3 PUFAs when compared to those who received a placebo [151]. Indeed, ω-3 PUFAs significantly inhibit the activity of NF-kβ, the master regulator of proinflammatory genes, which leads to the downregulation of IL1β and TNF-α, thus suppressing glial activation in APP/PS1 mice [140]. Similarly, MaR1 decreased the activity of Nf-kβ and chemokine secretion in human monocyte-derived microglia and human monocyte cell lines exposed to Aβ [144]. Furthermore, ω-3 PUFAs have been indicated to increase the production of brain-derived neurotrophic factors, decrease the production of proinflammatory cytokines, and induce anti-inflammatory microglial differentiation in human microglial cells [143]. Similarly, ω-3 PUFAs may help to resolve inflammation via their metabolites, called specialised pro-resolving mediators [152]. This evidence suggests that ω-3 PUFAs may improve the cognitive functions of COPD patients through the inhibition and resolution of inflammation.

In addition to their ability to promote Aβ clearance and modulate inflammatory processes, ω-3 PUFAs have demonstrated antioxidant properties. Indeed, a study has shown that treatment with ω-3 PUFAs remarkably attenuated increases in hippocampal malondialdehyde and 8-hydroxy-2′-deoxyguanosine levels as well as decreases in reduced glutathione (GSH) levels and the GSH-peroxidase activity induced by pentylenetetrazol kindling in young rat models [153]. Similarly, dose-dependent reductions in LPS-induced nitric oxide and ROS generation and inducible nitric oxide synthase expression have been reported in mice following treatment with krill-oil-derived ω-3 PUFAs [154]. Notably, the beneficial effect of ω-3 PUFAs on oxidative stress is related to their ability to enhance the expression of nuclear factor erythroid 2-related factor (Nrf-2), which is the master regulator of the antioxidant enzyme genes. Indeed, ω-3 PUFAs have been shown to improve rats’ antioxidant defence in astrocytes via the Nrf2-dependent mechanism, and this effect depends on the ratio of DHA/EPA that is incorporated into membrane phospholipids [155]. The activation of Nrf2 promotes the expression of key antioxidant enzymes, such as catalase, glutathione peroxidase, and superoxide dismutase, which increases the body’s resilience to oxidative stress [156]. A recent study has demonstrated that DHA directly activates Nrf2-signalling pathways, reducing the degree of oxidative damage caused by Aβ25–35 in PC12 cells [157]. In summary, owing to their antioxidant properties, ω-3 PUFAs may help to manage CI in COPD patients, thus mitigating the oxidative stress associated with COPD.

Despite the compelling evidence suggesting the promising potentials of ω-3 PUFAs in managing COPD comorbid with CI, interventional studies are warranted to test the efficacy of ω-3 PUFAs in this patient population. Meanwhile, ω-3 PUFA supplementation of up to 3.5 g per day in COPD patients is generally safe and well tolerated [149,158], and no severe adverse events associated with ω-3 PUFA supplementation have been reported [159,160]. On the other hand, the use of ω-3 PUFAs in managing CI in COPD patients may be limited by delayed therapeutic responses and suboptimal compliance. In particular, ω-3 PUFAs may take relatively longer than standard medications to bring about their intended effects. COPD is often linked with various additional health conditions. As a result, individuals with COPD are typically prescribed multiple medications to address these accompanying issues, leading to a high incidence of non-adherence to prescribed medications [161,162]. Furthermore, mood disorders, like anxiety and depression, are quite common in individuals with COPD and have been reported to impede adherence to medication regimens [163]. Therefore, we suggest conducting well-designed trials with sufficient follow-up periods (from several weeks to months) and appropriate measures to ensure compliance to assess the effectiveness of ω-3 PUFAs in managing COPD-related CI.

### 4.2. Parkinson’s Disease

Numerous studies have explored the relationship between ω-3 PUFAs and Parkinson’s disease, and the findings have been promising. A case-control study with a meta-analytic component reported a reverse correlation between Parkinson’s disease and the consumption of PUFAs, particularly ω-3 PUFAs or their precursor (ALA) [164]. Additionally, higher intakes of ω-3 PUFAs were associated with a reduced risk of Parkinson’s disease in a three-decade prospective cohort study [165]. Moreover, a 6-year study involving 5289 individuals found a significant link between the consumption of ω-3 PUFAs and a lower incidence of Parkinson’s disease [166]. A 16-year follow-up study with 131,368 participants attributed a lower risk of Parkinson’s disease to high intakes of fish, poultry, fruits, and vegetables [167]. Because all the above evidence stems from observational studies, a direct cause-and-effect relationship between the consumption of ω-3 PUFAs and the risk of Parkinson’s disease should be approached cautiously. Nevertheless, the evidence suggesting a lower incidence of Parkinson’s disease associated with ω-3 PUFA consumption is intriguing.

Currently, no clinical trials are specifically focused on ω-3 PUFA monotherapy for Parkinson’s disease, and those involving combination therapy are limited. However, the results from these studies are encouraging. In one clinical trial, Parkinson’s disease patients randomly assigned to the supplement group (containing ω-3 PUFAs, ω-6 PUFAs, and antioxidants) exhibited a delayed progression of Parkinson’s disease, as assessed by the Unified Parkinson’s Disease Rating Scale (UPRDS), after a 30-month follow-up [168]. Furthermore, Parkinson’s disease patients treated with ω-3 PUFAs (1000 mg/day) and vitamin E (400 IU) for 12 weeks reported improved UPDRS scores and lower levels of hs-CRP when compared to the placebo group [169]. Additionally, a 3-month daily supplementation with ω-3 PUFAs (1000 mg) and vitamin E (400 IU) led to an upregulation of the expression of the peroxisome proliferator-activated receptor (PPAR)-gamma gene and a downregulation of TNF-α gene expression in peripheral blood mononuclear cells of Parkinson’s disease patients [170]. In summary, the beneficial effects of ω-3 PUFAs on the progression of Parkinson’s disease, as indicated by low UPRDS scores and hs-CRP levels, have been partly attributed to the ability of ω-3 PUFAs to modulate inflammatory processes (see Figure 2). Indeed, ω-3 PUFAs have been shown to regulate inflammatory pathways by modifying the composition of cell membrane phospholipids, disrupting lipid rafts, suppressing the synthesis of eicosanoids from arachidonic acid, inhibiting the activation of NF-kβ, and activating PPAR-γ [171,172,173,174]. Despite the proven benefits of ω-3 PUFAs in managing Parkinson’s disease, studies on the effects of ω-3 PUFAs on comorbid Parkinson’s disease in COPD patients do not currently exist, to the best of our knowledge. Thus, studies are needed in this regard.

## 5. Conclusions and Future Prospects

COPD is associated with CI, leading to poor clinical outcomes, reduced compliance with treatment protocols, decreased quality of life, and increased mortality among the COPD populations. Despite its devastating effects on COPD patients, CI receives little or no attention in COPD management. Important mechanisms that could predispose COPD patients to CI include hypoxia, oxidative stress, inflammation, cigarette smoking, gut dysbiosis, and Aβ deposition in the brain. Interestingly, ω-3 PUFAs and their metabolites have been proven to modulate inflammatory pathways, activate antioxidant enzymes, and promote Aβ clearance from the brain. However, no evidence indicates a connection between CI in COPD patients and a deficiency in ω-3 PUFAs. Additionally, there is a lack of studies regarding the potential therapeutic benefits of ω-3 PUFAs in managing CI in COPD patients. This gap in research is partly because CI is a neglected issue in managing COPD, with only a small portion of COPD patients receiving treatment for CI. Therefore, studies are needed to investigate the roles of ω-3 PUFAs in CI associated with COPD. The outcomes of these studies will aid in designing interventional studies to assess the impact of these promising nutritional supplements on improving CI in the COPD population. These trials should also determine the appropriate dosage and formulation of ω-3 PUFAs necessary to enhance the cognitive functions of COPD patients. We hypothesise that ω-3 PUFAs will improve the executive functions of patients with COPD. This enhancement will enable COPD patients to effectively adhere to the protocols for managing their condition, resulting in improved clinical outcomes. However, as COPD is associated with several comorbidities, such as cardiovascular disorders, diabetes, hypertension, and mood disorders, managing these comorbidities along with COPD will result in a better quality of life for COPD patients.

## Data Availability

Data sharing is not applicable to this article as no new data were created or analyzed in this study.

## Abbreviations

Aβ	Amyloid-beta
ALA	Alpha-linolenic acid
BBB	Blood–brain barrier
CI	Cognitive impairment
COPD	Chronic obstructive pulmonary disease
CRP	C-reactive protein
CSF	Cerebrospinal fluid
DHA	Docosahexaenoic acid
EPA	Eicosapentaenoic acid
GSH	Reduced glutathione
HIF-1	Hypoxia-inducible factor-1
HRQoL	Health-related quality of life
hs-CRP	High-sensitivity C-reactive protein
IFN-γ	Interferon-gamma
IL	Interleukin
L-DOPA	Levodopa
LPS	Lipopolysaccharide
MaR	Maresin
NF-kβ	Nuclear factor kappa-beta
Nrf-2	Nuclear factor erythroid 2-related factor
PPAR-γ	Peroxisome proliferator-activated receptor-gamma
PR	Pulmonary rehabilitation
ROS	Reactive oxygen species
RvD	Resolvin D
TGF-β	Transforming growth factor-beta
TNF-α	Tumour necrosis factor-alpha
TSPO	Translocator protein
UPDRS	Unified Parkinson Disease Rating Scale
ω-3 PUFA	Omega-3 polyunsaturated fatty acid
